# Pediatric cervical spine injuries with neurological deficits, treatment options, and potential for recovery

**DOI:** 10.1051/sicotj/2017035

**Published:** 2017-09-06

**Authors:** Belal Elnady, Essam El-Morshidy, Mohamed El-Meshtawi, Ahmed Shawky

**Affiliations:** 1 Department of Orthopedic and Trauma Surgery, Assiut University Medical School 71111 Assiut Egypt; 2 Department of Spine Surgery, HELIOS Klinikum Erfurt 99089 Erfurt Germany

**Keywords:** Pediatric spinal injuries, Pediatric cervical trauma, Pediatric spinal cord injury

## Abstract

*Purpose*: The purpose of the present study was to highlight the challenges in managing cervical spine injuries in children with neurological deficits.

*Introduction*: Cervical spine injuries in children are relatively rare. Pattern, severity, and level of these injuries are age dependent. Neurological deficits in young children are uncommon and usually have a good potential for recovery.

*Patients and methods*: This report includes four cases with pediatric cervical spine injuries with variable degrees of spinal cord injuries and neurological deficits. All the four patients were five years old or younger at the time of injury. Those patients were presented with different patterns of injuries and the treatment was customized for every patient. Marked neurological improvement occurred in all patients at the last follow-up.

*Conclusion*: The treatment of pediatric cervical spine injuries should be individualized. Children with stable injuries should do well with non-operative treatment while operative treatment is recommended when the indication is appropriate and the expertise is available. Neurological deficits due to spinal cord injuries in pediatric patients have a high potential for recovery, provided that adequate management is considered.

## Introduction

Cervical spine injuries in children are relatively rare, representing only about 2% of all spine trauma [1]. Pattern, severity, and level of these injuries are age dependent [2]. Evaluation and clearance of cervical spine injuries in children is obviously a difficult job due to lack of cooperation during examination and imaging procedures.

Neurological deficits in young children are uncommon and usually have a good potential for recovery. However, it carries the risk of catastrophic permanent neurological disability [3].

## Case 1

**Figure 1.**
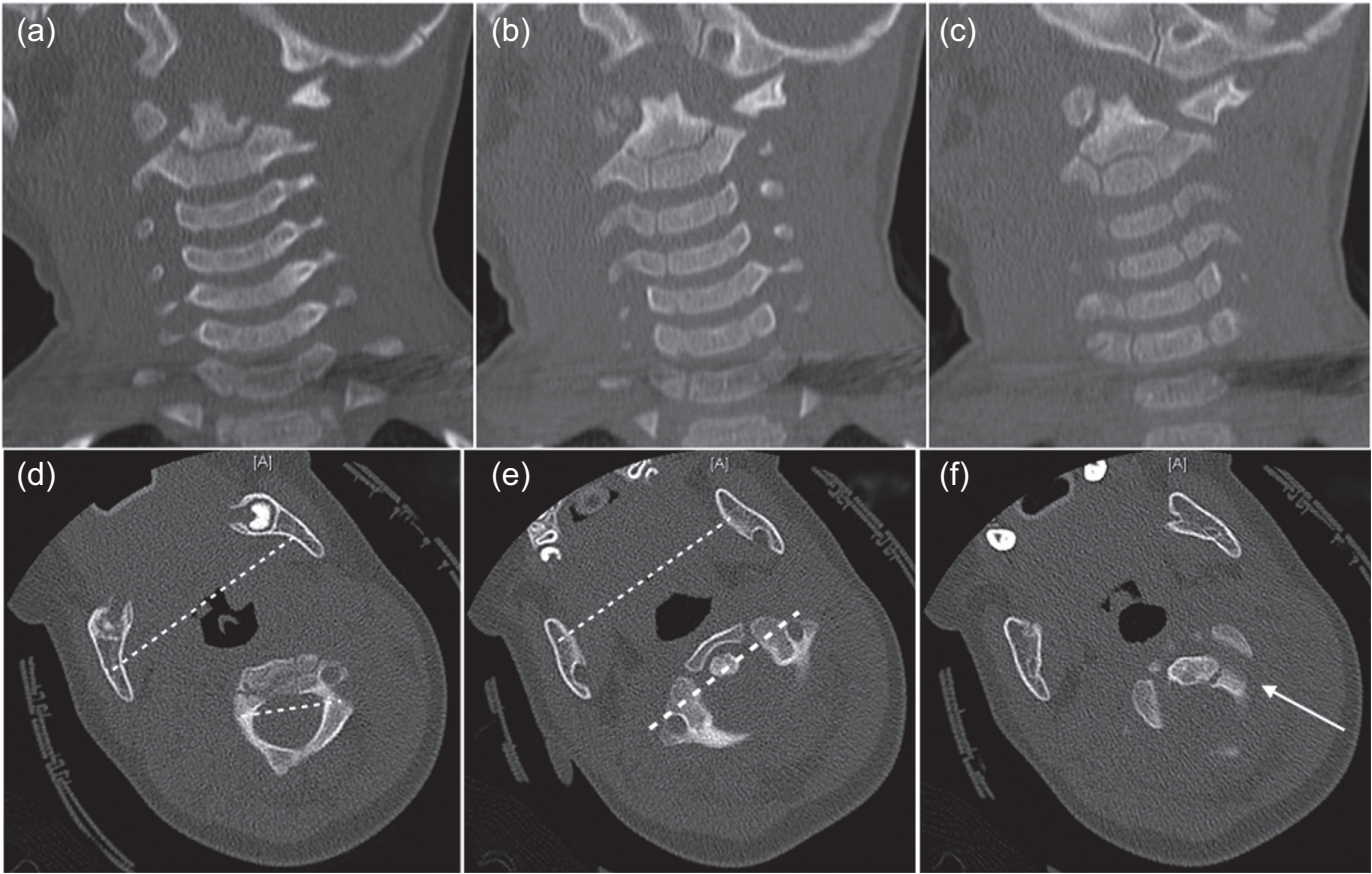
Case 1: A 16-month-old boy who sustained a motor vehicle accident. (a–c) Pre-reduction computed tomography (CT) scans showing the asymmetrical distance between dens and C1 lateral mass on both sides. (d) The difference between the transverse axis of the skull and C2. (e) The alignment of the transverse axis of C1 and skull. (f) The dislocated C1/2 joint on the left side (arrow).

**Figure 2.**
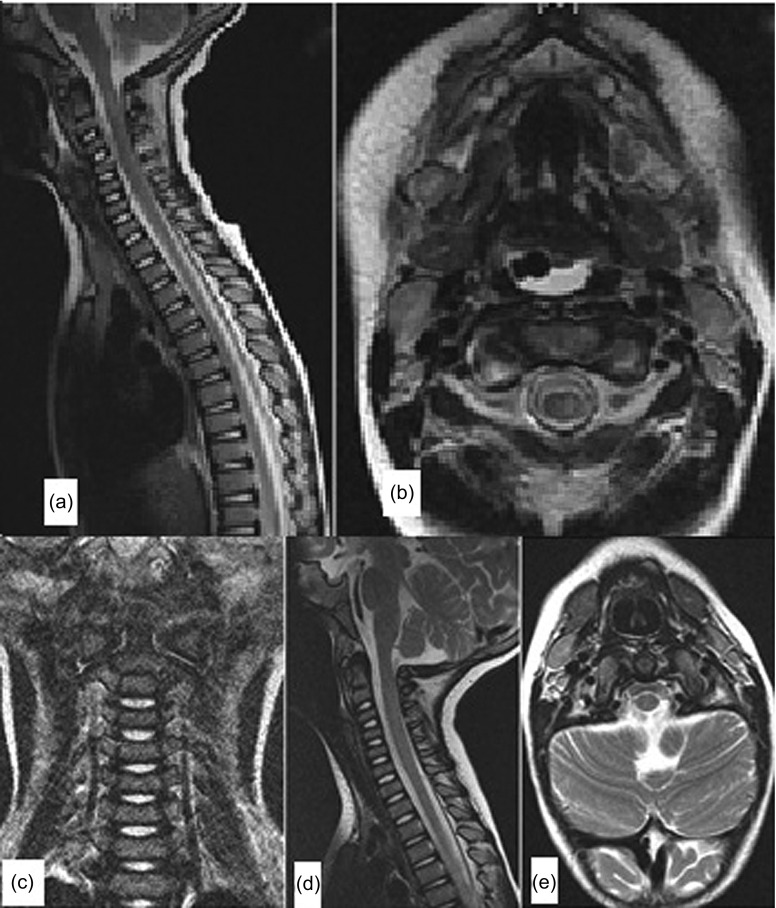
Case 1: (a, b) Pre-reduction MRI shows the disco-ligamentous injury at C3/4 level, and the spinal cord edema on the left side seen in axial cut. (c–e) Follow-up MRI shows the healing of the disco-ligamentous injury at C3/4 level, and complete subsidence of the spinal cord edema.

A 16-month-old boy presented to the emergency room following a motor vehicle accident. He sustained a traumatic rotary subluxation of C1/2 (Fielding type 2) combined with a C3/4 disco-ligamentous injury. Neurological examination revealed a left-sided hemiparesis. Under general anesthesia, closed reduction was achieved through controlled axial traction of the head under image intensifier to avoid over-distraction at the C3/4 disco-ligamentous injury. Then rotation of the head to the left completed the reduction with an audible click. Immobilization in a Minerva cast was done. Three weeks later, the cast was replaced with a customized Minerva orthosis, which was in place for another three weeks. The neurological deficit progressively improved such that the child was almost neurologically normal at six weeks follow-up. Follow-up magnetic resonance imaging (MRI) after three months showed complete regression of the spinal cord edema, reduction of the facet joint on both sides, and complete healing of the C3/4 disco-ligamentous injury. At six months follow-up, complete recovery of the neurological deficit was evident with painless normal movement of the cervical spine in all directions (Figures 1 and 2).

## Case 2

**Figure 3.**
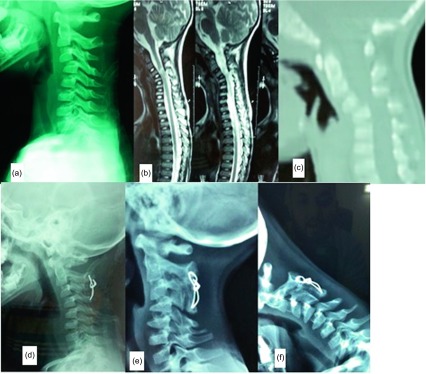
Case 2: A four-year-old boy. (a) Preoperative plain radiograph showing C3/4 subluxation, (b) preoperative MRI showing spinal cord edema, (c) preoperative sagittal CT scan confirming C3/4 subluxation, (d–f) one-year follow-up lateral, flexion, and extension X rays showing solid posterior C2–C4 fusion.

A four-year-old boy presented to the emergency room following a motor vehicle accident. Neurological examination revealed complete quadriplegia and radiological examination revealed C3/4 subluxation. MRI revealed extensive cord edema opposite to C3/4 levels. Under general anesthesia, closed reduction was tried guided by image intensifier to avoid over-traction. The reduction was unstable with resubluxation of C3/4, hence, the child was planned for operative intervention. Through a posterior approach, the subluxed C3/4 facet was reduced using a micro-dissector, then fixation was done using a cerclage wire through the spinous process of C2–C4 as there was an incidental fracture of the C3 spinous process. Decortication of C2–C4 laminae was done using a burr and then local bone graft was added. Postoperative X rays showed maintained reduction and a rigid collar was applied for six weeks. At the latest follow-up one year after surgery, the child was walking unsupported with mild spasticity in his legs with reasonable hand function. Radiological evaluation revealed solid fusion between C2–C4 (Figure 3).

## Case 3

A five-year-old boy was presented to the outpatient clinic with a history of a motor vehicle accident six months previously which resulted in traumatic C1/2 instability with incomplete quadriplegia. He had an operation in another hospital during which he had a non-instrumented C1/2 fusion using iliac bone graft and absorbable sutures. Neurological examination revealed Frankle C quadriparesis, while radiological evaluation showed persistent C1/2 instability with C2/3 posterior fusion (which may have resulted unintentionally from the previous surgery).

**Figure 4.**
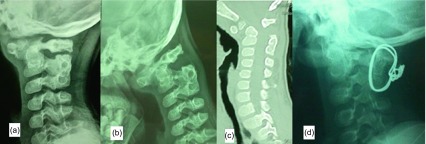
Case 3: A five-year-old boy. (a, b) Preoperative flexion and extension lateral cervical X rays showing C1/2 instability with clear pseudoarthosis and posterior C2/3 fusion, (c) preoperative sagittal CT scan showing anterior subluxation of C1, (d) postoperative X ray showing reduction of C1 with fixation of C1/2–3 with sublaminar cerclage wire.

This child was planned for revision surgery. Through a posterior approach, reduction of C1 was done using a blunt bone micro-hook under the C1 arch. Fixation was done using a sublaminar cerclage wire under C1 arch and the fused C2/3 laminae. Decortication of C1 arch and C2/3 laminae was done using a burr and iliac bone graft was added. A rigid cervical collar was used for six weeks. At six-month follow-up, the child’s neurological status was back to normal (Frankle D) and radiological examination showed solid C1/2–3 fusion (Figure 4).

## Case 4

**Figure 5.**
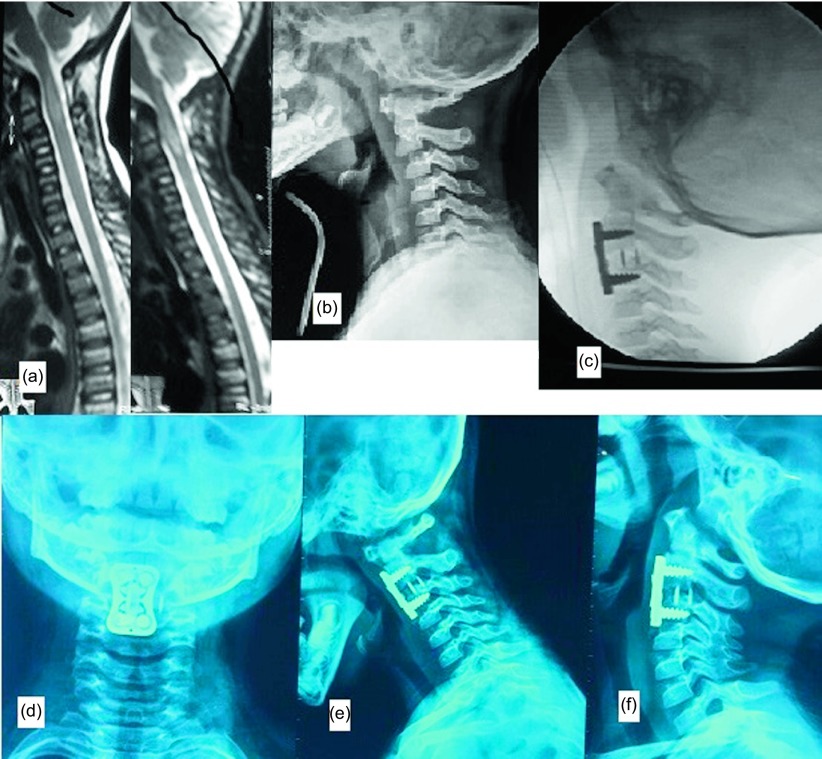
Case 4: A four-year-old girl. (a) Preoperative MRI showing disco-ligamentous injury at C2/3 level with spinal cord edema, (b) preoperative lateral X ray showing C2/3 subluxation, (c) intraoperative radiograph confirming the reduction of C2/3 with fixation using cage and cervical locked plate, (e–g) one-year follow-up anteroposterior (AP), flexion and extension lateral X rays showing solid fusion.

A four-year-old female child presented with neck pain and Frankle B quadriparesis with stable general condition following a fall from a three meter height. The plain radiograph showed C2/3 subluxation while MRI showed extensive spinal cord edema with disco-ligamentous injury at C2/3. Due to the marked instability of C2/3 levels in plain radiograph, anterior C2/3 discectomy and fusion using a cage and a cervical locked plate was done. At the last follow-up six months after surgery, the neurological status had improved to normal (Frankle D) with solid C2/3 fusion on the plain radiograph (Figure 5).

## Discussion

The treatment of pediatric cervical spinal injuries has traditionally been conservative. Unstable pediatric spinal injuries are increasingly being treated operatively [4–6]. Operative fixation in young children still has the challenge of appropriate implants and instrumentation.

Several fixation methods had been described for the fixation of pediatric cervical spine. This included wiring [7, 8], locked plates [6, 9], rods and wires [8], and even sutures [5]. In this report, cerclage wire was preferred as a simple and effective method for posterior fixation. Children have very rapid healing power, and fusion is expected in a short time with low risk of metal failure. A conventional anterior cervical plate was used for anterior stabilization, however, a careful measurement of the plate and screw length is important.

Conservative treatment has a significant role in the management of stable pediatric cervical spine injury. In a study of unstable upper pediatric spine injury by Duhem et al. [8], 21 of 28 children were treated conservatively. The indications for surgery were: persistent instability in spite of halo traction, neurological deterioration, and irreducible fracture-dislocation. Parisini et al. [10] noted that conservative treatment was successful in stable fractures but failed in unstable injuries. Mortazavi et al. [11] treated 36 of 48 patients with multilevel spinal injury non-operatively, none of the conservatively treated patients needed surgical intervention in the follow-up period.

The prognosis for recovery from spinal cord injuries in pediatric patients is much better than the adult population. Children have rapid healing and more regeneration power of the nervous system [3, 4]. All patients in this report had spinal cord injury of variable degrees with marked improvement of all of them at the last follow-up. Several authors reported on complete neurological recovery in children after traumatic cervical spine injuries [4, 8, 9].

## Conclusion

The treatment of pediatric cervical spine injuries should be individualized. Children with stable injuries would do well with non-operative treatment often involving the use of customized orthosis. Operative treatment is recommended for unstable injuries when the indication is appropriate and the expertise is available. Neurological deficits due to spinal cord injuries in pediatric patients have a high potential for recovery provided that adequate management is considered.

## Conflict of interest

All authors certify that they have no financial conflict of interest in connection with this article.
